# Comprehensive 5P framework for active aging using the ecological approach: an iterative systematic review

**DOI:** 10.1186/s12889-019-8136-8

**Published:** 2020-01-09

**Authors:** Azadeh Lak, Parichehr Rashidghalam, Phyo K. Myint, Hamid R. Bradaran

**Affiliations:** 1 Faculty of Architecture and Urban Planning, Shahid Beheshti University, Tehran, 1983963113 Iran; 2 Ageing Clinical & Experimental Research Team, Institute of Applied Health Sciences, University of Aberdeen, Aberdeen, UK; 3 Ageing Clinical & Experimental Research Team, Institute of Applied Health Sciences, University of Aberdeen, Aberdeen, UK; 4 Department of Epidemiology, School of Public Health, Iran University of Medical Sciences, Tehran, Iran

**Keywords:** Active aging, Integrative systematic review, Ecological model

## Abstract

**Background:**

“Active aging” is an inclusive term and has been defined from a variety of aspects in different domains throughout the literature. The aim of this review was to identify those aspects that play significant roles in building this concept using an ecological approach.

**Methods:**

In this study, seven online databases, including JSTOR, Pub-Med, Web of Science, Google Scholar, ProQuest, EBSCO, and Scopus, were searched from 2002 to 2018 for both qualitative and quantitative articles published in English. Two reviewers independently found the related articles using the search terms “active aging” and “built environment” and included both “ageing” and “aging”.

**Results:**

Of 1500 records which passed the screening stage, 92 were eligible for inclusion in the review. A total of 15 subthemes were derived: (1) personal characteristics, (2) behavioral attitude, (3) land use, (4) access, (5) physical form, (6) cityscape/city image, (7) public open spaces, (8) housing, (9) social environment, (10) cultural Environment, (11) economic environment, (12) good governance, (13) physical health, (14) mental health, and (15) social health. Ecological themes of active aging can be defined as the 5P model: person, processes, place, prime, and policymaking.

**Conclusions:**

The results of this study can shed light on different aspects of active aging. Also, the results emphasized the significance of the multidimensional nature of active aging, micro (person), meso (process), and macro systems (place and policymaking), based on health (prime) environments. Moreover, the results were based on the relationships between the person and the environment at the individual, interpersonal, and environmental levels, which can be used to conduct future studies and develop policies on aging populations.

## Background

Creating positive aspects of aging life is an important factor in achieving health expectancy. In societies with a growing elderly population, great attention should be paid to the participation of the elderly in their own well-being and that of their families. According to the UN (2015), the proportion of older people ≥65 years will skyrocket from 901 million (12.3%) in 2015 to 1.4 billion (16.5%) in 2030 (56% increase). Active aging is referred as aging well [1], and according to WHO (2002), the elderly will be able to sustain health and well-being if they increase their participation in daily activities. WHO has also described the goal of active aging as the process of optimizing opportunities for health, participation, and security to enhance the quality of life as people age, while noting that these policies and programs should be based on the rights, needs, preferences, and capacities of older people [2].

The societies which aim to provide opportunities for older people to take part in national schemes, including social security schemes, environmental and urban planning, health services, civil society, and legislation, are likely to reach the goal of active aging. Active Aging Index is the means to rank different countries based on their status in such societal measures as the participation of the elderly in the workforce or life expectancy [3]. This is perhaps why WHO (2002) does not interpret active aging as a highly standard quality of life for a group of people, because this term is not considered as a phenotypic description of an individual or individuals.

However, the term active aging has been used to refer to different aspects in recent years [3]. For instance, several researchers classified and offered a definition of the active aging phenotype according to WHO: good functional ability and fitness; continued involvement in one’s family and/or peer group; maintenance of positive subjective well-being; a good physical, social, and mental health; and engagement with community throughout the aging process. These factors have been proposed as key aspects that describe an active aging phenotype [4, 5].

There are several ignored ecological aspects that are considered to be conducive to the concept of maintaining active aging communities. Therefore, this study aimed to explore the topic with a new approach to analyze the determinants of active aging through a narrative review. The ecological approach considers aging as an interplay between an individual’s functional age and adaptation with the physical and social environment [6], which links aging to the respective concepts of urban design and service planning for disability and aging. Such approaches are wide-ranging, including the creation of healthy cities, livable communities, walkable communities, universal design, and accessibility [7, 8]. Although all these notions aim for different goals, they commonly provide older people with essential elements for health: (eg, accessible and affordable health and healthcare services, opportunities to stay active, etc.), social security (eg, home and pedestrian safety, neighborhood safety [9], community safety, transportation safety, financial security, affordable housing, and services, etc.), which allow active social participation and engagement activities (eg, through accessible public transportation, information services, recreational programs, social connections, volunteer opportunities, and places to worship, etc.) [10].

Thus, cities and urban environments should focus on their local conditions aiming for the health and comfort of the older people while acknowledging their impact. Cities, enjoying their long-time experience of working with local communities and local problems, are also in the right position to satisfy the needs of aging adults [11–13]. To this end, there is a need to identify the factors that contribute to different aspects of health in older people while understanding the elements that could prevent them from taking part in daily activities. Also, mobility and independence, which may lead to a lower level of assisted living conditions and dependency, are of significant importance [12–14].

A review provided an analysis of research evidence according to the proposed questions with a specific systematic method to determine, select, and appraise the related primary research [15]. Therefore, in this narrative review, the aging population was defined as those who are 60 years or over and seek to respond to cultural and national differences. In this study, it was aimed to offer an understanding of what components of the activity of the elderly, built for the elderly in the environment based on the ecological perspective, can provide the opportunity for further studies on active aging.

## Methods

This was a narrative review of a series of studies on the topic of active aging [16]. This led the authors to decide on the classification of the experiences, social contexts, and views on active aging as a common theme based on the ecological approach of the related articles [17–19]. Therefore, theoretical and empirical studies were also analyzed to merge (synthesize) the data as a narrative review [20]. In line with the existing literature, the aims of the study were as follow: defining concepts, reviewing evidence, analyzing the methodological issues of the concept, and reviewing the theories [21].

### Search strategy and study selection

**Fig. 1:**
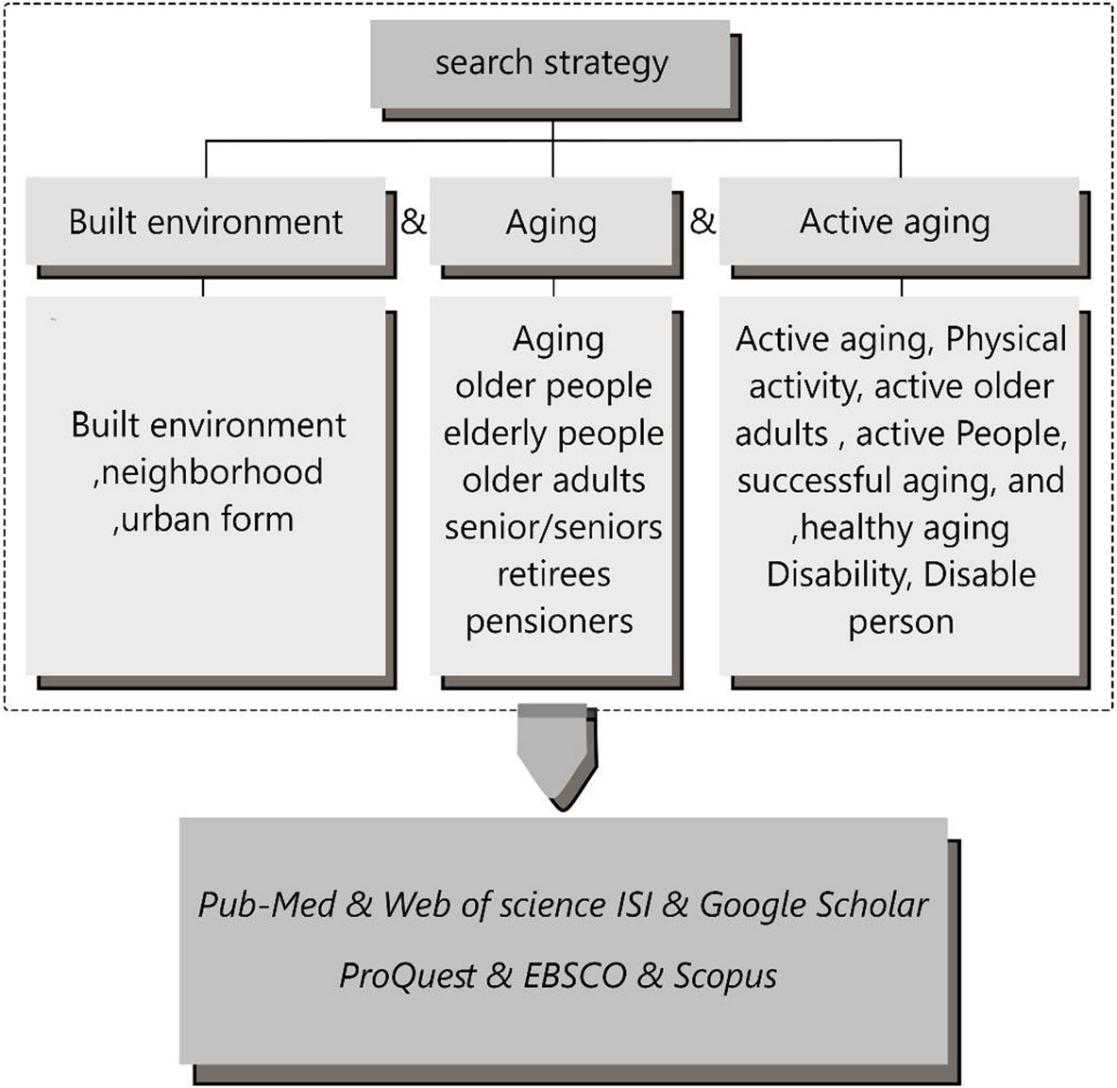
Search Strategy Summary With Keywords

An iterative approach was used in this study as a narrative review and allowed the authors to revise the inclusion and exclusion criteria (Table 1), search strategy (Fig. 1), and the main research questions after considering the evidence [20, 22]. Problem identification stage clarified that although aging is a natural part of life, active aging is a positive concept compared to inactive aging (living in a nursing home). Therefore, studying active aging also includes investigating the aspects and characteristics of the aging potential and offering methods to improve the understanding of aging [23]. The main question of this research was *“What are the effective attributes in developing the notion of active aging according to the ecological model?”*

**Table 1:** Inclusion/Exclusion Criteria for Selecting the Articles for This Review

Inclusion	Exclusion
- Sampling of community-dwelling older adults aged 60 years or older; assessing health-related issues or component behaviors of the World Health Organization’s active aging concept; studies that considered the environment or related concept as a potential correlate of health or activity; and studies that adopted qualitative, quantitative, or mixed methods - Application of a stated theory or conceptual framework - Papers with English abstract or summary	- Participants were not only from residential environments - Not limited to residential properties only

The literature search was done as the second stage of narrative review from August to October 2018 and updated again in January 2019. A total of 7 online databases (Pub-Med, Web of Science, ISI, Google Scholar, ProQuest, EBSCO, and Scopus) were searched. The keywords used were “active aging” and “built environment” and included “ageing” and “aging” “senior”/"seniors”, “retirees”, and “pensioners”.

**Fig. 2:**
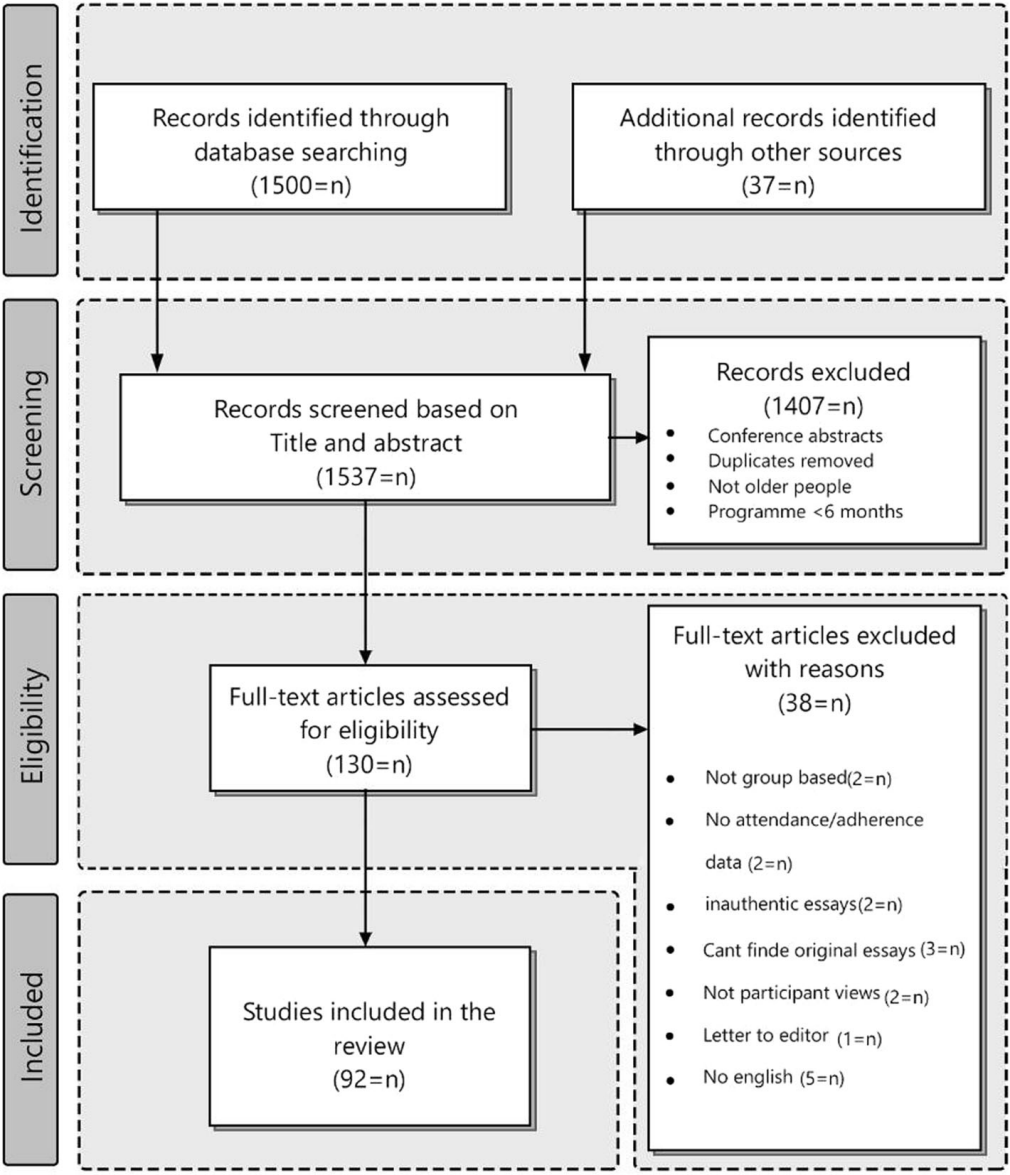
Flow Chart of Study Selection

To provide an inclusive search strategy, a common review strategy of building blocks was applied and search items were categorized into concepts and later expanded with the synonyms through Boolean operators [20]. Berry Picking, which is commonly applied in the iterative search and allows the search strategy to evolve from the information obtained throughout the review process, was also used [24, 25]. Whenever a piece of new evidence was discovered, this review approach allowed the modification of the strategy based on the new evidence. In addition, the drop a concept searching technique allowed the stacking of terms approach to be used by firstly combining all term/concepts of the review and then removing the least relevant concepts to cast a wider search net [20]. Inclusion criteria have been adopted based on the Boolean strategy, which included “active ageing”/“active aging” in the title and in the abstracts with the following terms: “model,” “definition,” “theory,” “structure,” “dimension,” and “attributes”. Then, after collecting the full-text studies, some terms were excluded to avoid overlapping, eg, aging, healthy aging, successful aging, and aging well. The key searched terms were classified into the following categories: (1) active aging (older people, elderly people); (2) built environment (built environment, housing, and building capacity); and (3) health outcomes (mental health, physical health, social health, wellness, well-being, disability, quality of life, comorbidity, functional limitations, disabled persons, and mentally disabled persons) (Fig. 2).

### Data extraction and quality assessment

The results were recorded in a reference manager database and the titles and abstracts were screened by the main reviewer (AL). The team members verified the records with respect to their rigor and completion through secondary blind screening of 30% of the original 1500 records. Then, studies that met the inclusion and exclusion criteria (Table 1) were again checked and regular meetings were held to resolve the disagreement, if any, and discuss the review process.

### Data analysis

Narrative synthesis, along with qualitative content analysis based on mixed inductive and deductive approaches, was used for data analysis through identifying the themes emerging from the evidence [17, 18]. The steps of qualitative content analysis were organized according to Renz et al. (2018), which included “(a) preparing the data, (b) reading transcripts repeatedly to achieve immersion and obtain a sense of the whole, (c) making notes on the transcripts listing the different types of information found in the text, (d) defining the unit of analysis using themes as the unit of analysis versus linguistic units, (e) developing a coding scheme to organize data in a comprehensible way, (f) coding all the texts, (g) making conclusions from coded data, and (h) describing and interpreting the findings [26].

The aim of this study was to investigate the current body of research on ecological aspects of active aging. As defined by WHO, active aging includes the following attributes ( [27], 1) autonomy: controlling, coping, and making personal decisions based on personal rules and preferences, (2) independence, the ability to perform functions related to daily living—that is the capacity of living independently in the community with no and/or little help from others; and (3) quality of life: an individual’s perception of their position in life in the context of the culture and value system where they live and in relation to their goals, expectations, standards, and concerns. Active aging, as a broad-ranging concept, incorporates a person’s physical health, psychological state, level of independence, social relationships, personal beliefs, and relationship to salient features in the environment [28]. As age increases, the quality of life the person becomes mainly dependent on their independence and autonomy and their healthy life expectancy. Positive subjective well-being, continued involvement in one’s family, peer group, and community, good physical, social, and mental health, and good functional ability and fitness are among the components recognized to define active aging [4, 5].

The concept of active aging is the result of several components which help to identify the factors that act both as risks and supporting elements of active aging. The ecological approach is a general approach to geriatrics which focuses on multiple effective levels and helps to increase the level of physical activity in the total population, particularly the elderly as a separate group [29]. The proposed model provided the authors with an inclusive approach to understand the effective factors on physical activity based on which influential interventions could be offered for behavioral change [30].

Figure 2 shows the study selection process and exclusions. The search identified 1500 studies, of which 92 articles were eligible to be included in this review. Also, 48 articles included quantitative and mixed methods, while the remaining 45 studies applied qualitative methods and reviews. The 2 coauthors (AL and PR) independently performed data extraction, theme identification, and narrative summarization. Moreover, discussions with the other coauthor (HB) led to resolving discrepancies. Data synthesis began with an initial narrative evaluation of study characteristics and was completed with data reduction and comparison (Table 2).

**Table 2:** Themes of Active Aging Extracted From the Narrative Review

Themes	Sub-Themes	Codes	Definition	References
Person	Personal characteristic/determinants	Age		[7, 23, 31–34]
		Gender		
		Education level		
		Ethnicity		
		Residential tenure		
		Marital status		
		Home ownership		
		Household size		
		Current driving license		
		Employment		
		Eating and drinking habitat		[23, 35]
		Family support		[23, 36]
		SELF-CARE		[23, 37]
		SELF-PROMOTION		[23, 37]
		Mutual-help		[23, 37]
		Self-esteem		[23, 37]
		Life satisfaction		[23, 38]
		Travel behavior		[23, 39]
	Behavioral attitude/ determinants	Cigarette smoking		[38] [23, 40]
		Alcohol consumption		
		Practicing exercises/ kind/frequency/length of activity		
Place	Land- use	Shopping and obtaining services	The arrangement of activities and the the impact between trip origin and destinations Amount of activity in a given area The proximity of different land uses	[23, 33, 41–43]
		Service proximity		[23, 44]
		Public facilities		[23, 33, 45]
		Land use mix diversity / land-use composition	Amenities and facilities, such as library, community center, local shops, traditional clinics, community outreach projects	[33, 41, 43, 46–49]
		Facilities management		[50]
		Exercise, sports, and recreation facilities		[51, 52]
	Access	Connectivity	Connectivity and inter-linkages: Layering and sequence from private zone to community gathering zone and neighborhood Directness and availability of alternative routes through a neighborhood Directness and availability to different areas in a region, composed of street system, sidewalk network, pedestrian volumes, and directness of route	[43, 53–55]
		Accessibility services	The proximity of the home block and its neighborhood amenities Systems that provide connections between activities	[7, 23, 33, 48, 54, 56–63]
		Physical activity/ walkable environment/	Pavements and roads; safe pedestrian crossings Pedestrian infrastructure, good sidewalks, surface area of open space,	[31, 33, 41–43, 48, 49, 51, 53–56, 58, 59, 63–77]
		Mobility	Exterior and interior accessibility Ease of activities, convenience, disabled facilities, comfortable movement	[39, 52, 53, 55, 58, 65, 71, 78–81]
		Transportation (public)	Adequate and affordable public transport; bus stops	[7, 33, 57, 58, 60, 68, 71, 75, 79, 82]
	Physical form	Neighborhood characteristics	The number of noticeable differences in a street; also defines the level of the complexity of an environment, and, thus, the interest in the pedestrian	[43, 62]
		Urban Block: density	Lack of nuisance, free from crowds	[43, 83, 84]
		Safety: Traffic/speed, volume		[7, 31, 33, 41, 48, 63, 68, 75]
		Security: Crime/personal security/fall prevention architectural elements	Perceived safety, access to protection, environmental support, close environment satisfaction, care, and support from family, social support and Medicare	[7, 41, 42, 44, 53, 54, 60, 85, 86]
		Access to nature and green spaces	Contact with nature, green spaces, parks, gardens, micro-climate	[53–55, 79, 87]
		Topography / slope		[23]
	Cityscape/City Image	Perceived distance		
		Legibility/image	Way finding, understanding, and legibility of directions	[64]
		Perceived aesthetic/environmental attractiveness	Attractiveness and appeal of a place	[31, 33, 43, 46, 48, 55, 63, 75, 85]
		Natural scenery		[33, 41]
	Public open spaces	Street lighting	Outdoor lighting	[33, 53, 57, 88]
		Pedestrian safety		[9]
		Area of green and open spaces		[89]
		Recreation/ public open spaces		[90]
		Cleanness/lack of littering/vandalism/decay	Physical comfort: Cleanliness, visual attractiveness,	[43, 54, 55, 75, 85]
		Sufficient maintenance and management	Maintain structural and planting quality, upkeep of scenic beauty	[54, 64]
		Pollution (air, visual, noise, litter …)	fresh air, free from noise and congestion	[56]
		Pleasant environment		[54]
		Landscape	Outdoor seating/urban furniture/ spatial setting Seating area for rest, communal spaces, special seating, talking Spaces/	[43, 53–55, 57, 64]
	Housing	Universal design/ Housing quality variable		[47, 68, 91, 92]
		Neighborhood Safety		[9]
		Residential density/density of housing		[41, 47–49, 93]
		Older Residential Care Facility		[70]
		Outdoor gardens		[56, 94]
		Type of housing		[95–97]
Process	Social Environment	Life expectancy		[44]
		Quality of life / wellbeing		[1, 35, 52, 58, 70, 72, 93]
		Social interaction/ network	Community and social participation/interaction/relation, sense of community, community building, sense of belonging	[7, 14, 54, 60, 79, 98]
		Happiness		[99]
		Affordability		[44]
		Social inclusion	Ability to participate in economic and social activities (paid/volunteer work)	[41, 44, 53, 60, 63, 79, 83, 100]
		Social inequalities		[69]
		Social demography		[31, 101]
		Social democracy		[41, 102]
		Participation (in the planning, implementation and evaluation process, civic participation)	The sense of community ownership and involvement in site planning and management, social activities	[7, 32, 39, 42, 65, 79, 80, 83, 86, 103–106]
		Social class		[23]
		Social support/ community life facilities and services		[40, 51, 54, 63]
		Education, learning, employment and volunteering,		[40]
		Social capital/ social trust/ Social cohesion		[63]
	Cultural Environment	Religious activity		[42, 53, 57, 80, 85, 87]
		Cultural events/rituals/social activity	Forms of recreation, such as walking and other exercises	
		The sense of place: place attachment/ place identity	Heritage, sense of place, the importance of local identity, cultural components integrated into the planning and management of the site	
	Economic Environment	Health care services		[7, 40, 57, 86]
		Limited income/pension		[40]
		Insurance coverage		[40]
		Socioeconomic status		[31, 41]
		Affordable housing		[7]
		Car ownership		[41]
		Economic security		
		Homeownership		[1]
		Household income		[23]
		Living situation		[23, 41, 70]
		Employment		[23]
Policy Making	Good Governance	Effective collaboration and political commitment to the elder		[91, 107–113]
		Performance orientation	Managers /Independence and autonomy /Local Policies Planning and Governance	[61, 70, 73, 112–114]
		Openness, transparency, and integrity governance		
		Equity / inclusiveness		
Prime	Physical Health	Disability		[39, 52, 66]
		Public health / health environment	The sense of health, emotional well-being, relaxation and avoiding distress, happiness	[23, 39, 42, 53, 57, 67, 77, 88]
		Incidence of disease		[23]
		Pain feeling		[23]
		Functional ability		[23]
		Risk of institutionalization		[23]
		Self-reported falls		[23, 34]
		Self-reported health		[23]
		Physical activity		[1, 23, 42, 86, 93]
		Activities of daily living		[23]
		Genetic factors		[23]
		Body mass index obesity		[23, 67]
		Sleep hygiene		[37]
		Personal hygiene		[37]
	Mental Health	Depressive symptoms	Personal esteem, autonomy, and empowerment, independence, self-efficacy, attachment to the place from stress; PE: positive emotions; AC: attention capacity; CC: cognitive capacity.	[23, 39, 57, 65, 88, 93]
		Cognitive functioning		
		Psychological distress		
		Psychological wellbeing		
		Anxiety		
		Anger		
		Restorative activity		
		Spiritual activity		
		Self-actualization	Provide opportunities for learning, gaining knowledge	[53, 79, 115]
	Social Health	1) family, (2) work, (3) community involvement, and (4) social life		[39, 41, 70, 116, 117]
		sense of community identity; CE: community empowerment; SC: social capital; CL: culture		[98, 116]

### Identification of studies

Qualitative and quantitative data were extracted from one of the articles with mixed method (Barnett et al. (2017. To use the iterative search approach, some other articles underwent screening for key authors searches, reference searches, and citation searches. Next, the full-text studies were analyzed based on the inclusion/exclusion criteria (Table 1). Then, the lead author (AL) organized the data extracted from each study into larger subthemes and themes and other members of the research team verified the process.

## Results

A matrix was offered which included an outline containing the year, population, country, research method, and aspects of active aging concept (Appendix 1 and 2). The majority of the articles have been conducted during 2002 to 2018 and only a few have recently been published (eg, Ko & Yeung (2018)). Also, most of the articles were conducted in the U.S., and most of the quantitative studies used either surveys or second data analysis and follow-up methods. However, most frequently, quantitative studies focused on social engagement, physical, and mental well-being and built environment, while qualitative papers emphasized life satisfaction. Figure 2 demonstrates the flow chart adapted from preferred reporting items [27, 118].

### Thematic analysis

**Fig. 3:**
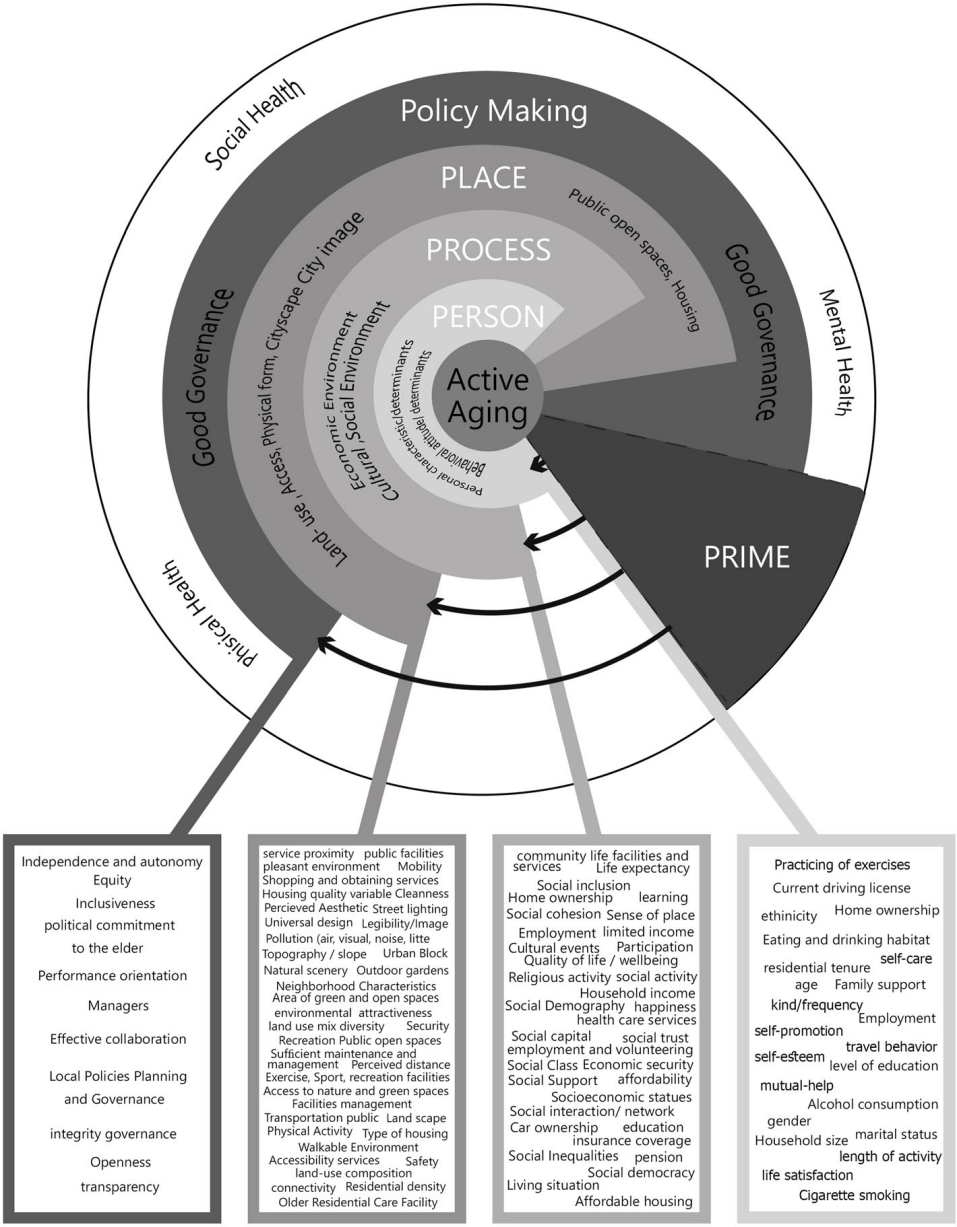
5P Ecological Model of Active Aging

Different aspects of active aging based on qualitative content analysis were coded and categorized during the process of data analysis, the results of which are shown in Table 2 in the form of codes, subthemes, and themes. Different aspects have also been presented and summarized in a matrix, with 15 subthemes and 5 themes called 5P model: (1) person (personal status), (2) process, (3) place (built environment), (4) policymaking, (5) and prime (Fig. 3).

### Themes

#### Person

One of the core themes is *“person”* that can be divided into 2 subthemes: personal characteristics and behavioral attitude. Most studies conducted on active aging provided an analysis of the effects of personal aspect, including health, age, shared genetic attributes, educational level, socioeconomic status, ethnicity, self-efficacy, and exercise history [23]. In addition, many studies included diet and lifestyle factors which are related to the person’s behavior such as adoption of a balanced diet and food restrictions [38]. These restrictive diets and eating habits appear to be aimed for a balance between the imposition of the aging physique and the limitations due to disease and sickness. Therefore, to maintain active aging, a healthy diet should be considered as a major component for older people to provide them with strong levels of health and well-being, which in turn help them experience growth and maturity [38]. Another component associated with the behavior aspect that can maintain active aging is the attitude towards these behaviors, because it can be modified at any point leading to a considerable increase in active aging [38]. Therefore, several studies found that those elderly who have never smoked or drank enjoyed a considerably better active aging compared to those with such habits [38]. Evidence also suggests that physical activity is a significant factor in active aging [23, 38].

#### Prime

The ecological model described by Stocks in the context of healthy environments is a broad framework which takes into account the physical environment and the psychosocial environment [119]. The proposed ecological model demonstrates the functional relationships between the aging person and the micro, meso, and macro system levels of the environment, along with the unstable equilibrium between environment and individual competence in very old age [118].

In this narrative review, “prime” is a reflection of health and components within the concept of health; namely, physical, mental, and social health. According to WHO definition, health is a state of complete physical, mental, and social well-being and not merely the absence of disease or infirmity [120]. This shows that several factors should be considered to grasp the idea of health, including biological, social, and psychological aspects.

The environment includes the natural and the man-made components alongside each other, which significantly affects the health state of an older person. Strong evidence supports the fact that living in an environment of low quality results in decreased physical health, associated with high prevalence of degenerative disease, incidence of falls, cardiovascular mortality, and reductions in longevity and increased disability, poor quality of life, and poor self-reported health [23, 42, 53, 54, 57, 67, 77, 87, 88]. Several mental health issues were also examined in the literature, including depression, anxiety, anger, psychological well-being, and cognitive functioning (mental processing, speed, and working memory) [23, 53, 57, 65, 79, 87, 88, 93, 115]. Social health [98] and well-being literature, on the other hand, focuses on the quality of interpersonal relationships between the members of a society and the amount of their involvement in their community. Several studies conducted on social well-being were assessed, and as a result of which it was found that to propose the level of social well-being for a group of people, their performance must be studied in the following areas: (1) family, (2) work, (3) community involvement, and (4) social life or sociability of individuals (eg, friendships) [117].

However, community health and psychological health are the results of the efforts contributed by epidemiologists and environmental psychologists, which include the sense of community identity, community empowerment, social capital, and culture [116].

#### Process

“Process” as the core ecological theme in active aging includes 3 subthemes: social, economic, and cultural environments, which are significantly important in the lives of an aging older person, as these can be barriers for life activities and may have health outcomes [23]. In this study, the literature search focused on those activities in the active aging policy framework by WHO that outlined key items as physical, social, cultural, civic, spiritual, and economic activities [2]. Many researchers have decided to study the role of social environement, which includes proximate social networks, social capital (norms of reciprocity and trust), and incidental social interactions [23]. Also, important components of social environement that affect an increased active aging include different social contacts, increased social involvement, wide social network, living children, ethnic homogenity, increased sense of neighborliness, increased literacy, increased social and economic status, increased workforce involvement, time spent with friends, and an age-specific community [1, 7, 23, 31, 32, 35, 40–42, 44, 51–54, 58, 60, 63, 65, 69, 70, 72, 79, 80, 83, 87, 93, 100–106, 121].

There are some specific characteristics of the social environment that requires the elderly to meet specific demands leading to suboptimal active aging. These may include family’s financial problems, a partner with health problems, unrealistic expectations of the person from their friends and families, and weak social and economic status of the area which is recognized as economic environment [1, 7, 23, 31, 40, 41, 70, 78, 86]. In addition, there is also the cultural environment which consists of religious activity, cultural events/rituals/social activity, and sense of place [42, 53, 54, 57, 80, 85, 87]. In this study, based on strong evidence, it was found that a powerful and supportive social network enhances the well-being and longevity of the elderly in the society. However, the composition of this supportive network may differ from one society/person to another [23]. The extent of poverty is also among the commonly mentioned factors that affects the activity involvement of the people. Yet, several studies maintained that lower social and economic status of an area is linked to the physical activity of individuals, which may be the case due to this group’s need for work and transportation. In fact, active aging includes social, cultural, civic, spiritual, and economic elements, which potentially contribute to health and well-being in later life [2, 23].

#### Place

The theme of “place” consists of land use, physical form, housing themes, access, public space quality, and city image/townscape. There are several reported environmental characteristics that enhance the elderly’s well-being, including the proximity to and density of public open space and recreational facilities, high-quality facilities (social and leisure facilities, age-appropriate facilities), peacefulness, cleanliness, safety of public areas and street crossings, frequent rubbish collection, access to health services, transport availability, closeness to shops and places for walking, living in a retirement village, living in a hillside area, living in an area with high rainfall and living in a residential environment [23], and lack of littering/vandalism/decay [40].

Furthermore, those studies that have addressed the importance of place in the discussion of active aging were identified, which included the following factors: measures of land use characteristics (area deprivation or poverty, and neighborhood degradation) [23, 33, 41, 42, 44–51]; physical form (neighborhood degradation, accessibility to services and facilities, accessibility of public greenspace, walkability, or pedestrian friendliness) [3, 7, 23, 31, 33, 41, 42, 48, 49, 51–79, 81, 82, 122]; physical form as security of perceived crime and antisocial behavior; safety of traffic conditions [7, 31, 33, 41, 42, 44, 48, 53–55, 60, 62, 63, 68, 75, 79, 83–87, 123]; quality of public space [23] as aesthetics and architecture, landscape (lighting and furniture) [33, 53–57, 64, 75, 85, 87–90], pedestrian-friendly features and availability of benches/sitting facilities [40]; trip hazards at home and neighborhood; home and environmental adaptations, climate and topography [23]; and favorable physical attributes such as trees and green areas which provide a sense of well-being and support resilience. Other positive factors were favorable street design, access to public transportation system, and several retail outlets which could be a motivating factor for more community involvement and physical activity [10, 33, 53–55, 57, 64, 75, 85, 87–90]. Identifying such diverse types of environments for careful analysis helps to assess homes or care centers and typology of land use, including rural and urban uses, categorization according to population density, defining areas based on time/distance, and defining a neighborhood by its members. Interestingly, in this review, it was found that most studies provided researcher-based definitions of a place as the built environment [23, 41, 47–49, 56, 68, 70, 77, 91–97].

#### Policymaking

Studying environments in the previous body of literature also included government-defined administrative areas as census tracts or postal codes [23], which focused on the key concepts of tolerance, fairness, social justice, and good governance (effective collaboration and political commitment to the elder performance orientation, openness, transparency, and integrity governance, and equity/inclusiveness) [61, 70, 73, 91, 107–112, 114, 124, 125] as necessary elements in sustainable development of urban planning. While physical and social environments are both important aspects to ensure the elderly’s health state, favorable governance and planning of the environment are of significant importance in building an age-appropriate community. To this end, several models have been proposed to address this important issue. However, the results showed that consensus planning using meaningful community involvement is significantly needed for these complex areas. Moreover, collaborative governance efforts with different players and sectors through the stages of building a community are also of great importance, as the public was considered as a body to consult with rather than active members in developing knowledge, space, or governance centers [126].

### Study quality

A considerable number of the included articles clearly provided objectives and methods compatible with the aims of their research. Comprehensive details of the studies’ quality are provided in appendices 1 and 2. In brief, since many of these articles applied a self-selected sampling, their results were enormously influenced by selection and information bias, which could affect their internal validity and, perhaps, the quality of quantitative studies, and to a lesser extent, qualitative studies. Furthermore, there were some observational studies which could not be considered as transferable and generalizable due to their small sample size and the methods used. This review was based on a qualitative process considering different aspects, which can provide different requirements for international scales, different types, etc. An iterative review, including all relevant literature to derive themes to accommodate smaller qualitative pieces of work that may have specific implications to international communities, small or large, with diverse societal, cultural, and religious belief systems, was conducted.

## Discussion

This narrative review was systematically conducted with the aim of defining the concept of active aging based on the ecological model. According to the findings, a 5P model, with 5 themes, including person, prime (health), process, place, and policymaking and 15 subthemes, was developed as the main framework of the ecological model of active aging. This framework provides the notion of the concept of active aging as a multidimensionality, multilayered (environment), and contextual concept from an ecological standpoint. Several results were obtained, and the first of which included specifications of the aging sample of the previous research most of whom were 65 years. However, based on WHO (2002), the old age is defined as 60 years or older. In addition, the age of the participants in different studies varied considerably, which made data comparison impossible, and there was also a lack of a mutual base for a functional definition of this concept.

The multidimensional concept of active aging does not merely focus on the physical activity of the older person alone, but it also consists of individual, social, and physical components, policymaking process, and environments with regards to physical activity, health, and the context in which such activities occur. In fact, this is in line with the previous body of research considering active aging as a multidimensional concept [23, 42]. According to the multilayered environmental nature of active aging, the findings of this study represent a notion that begins with an individual layer (person) and includes individual characteristics and behavioral attitudes, social or physical environment layer, and policymaking environment. However, these factors are closely linked together and all elements need to function harmoniously to achieve active aging in the context of societal, cultural, and religious belief systems. Indeed, this concept represents an ecological model [45, 127, 128] which focuses on the relationships between the environmental levels of the aging person within the 5P model, taking into account the micro (person), meso (process), and macro systems (place and policymaking) based on health (prime) dimension, along with the unstable equilibrium between environmental and individual competence in old age [118].

Active aging with contextual nature as a cultural and social notion [129] has its roots in diverse environmental contexts to clarify the ways a person reacts to and interacts with the environment they live in [128, 130]. According to Baltes’ theory of lifespan development (1987), throughout life, development always consists of the joint occurrence of gain (growth) and loss (decline) [49]. Considering the findings of this narrative review, active aging is a fruit of both personal and sociocultural environments which are strongly linked to the themes of policymaking and place according to the definition of health (prime). Perhaps the most important fact to be considered is that the aging population should be equipped with the necessary support to maintain an equilibrium between their decreased physical ability and increased transcendence, which is significantly obtained through access to personal, environmental, and social resources. This equilibrium includes physical and mental health at the micro level (personal), social well-being, and spirituality/transcendence (process) at the meso level, while living in a favorable and appropriate environment. Figure 3 demonstrates the details on policymaking that can ensure effective active aging.

This review was the first interdisciplinary and multidisciplinary study to define active aging. As active aging is a vast concept, it is essential to provide a multidisciplinary approach which covers its different aspects. Also, focusing on the elderly’s needs, including the need for active life with healthy behaviors results in their long-term positive outcomes which are of low cost and high value [131]. Moreover, to reach such a goal, the authors defined active aging as trying to maintain the components of health through participation in activities consistent with the individual’s objectives, abilities, and opportunities using the ecological model, which include 4 pillars of active aging for the elderly: goals in life, abilities, opportunities, and activities. In this narrative review, it was found that although the concept of active aging enhances the health status of individuals [132], the concept is faced with a few methodological issues. This methodological issue included the heterogeneity of the final studies and mapping factors of active aging, while focusing on the built environment. However, this review aimed to explore the socioecological approach which is motivating enough to create lifestyle changes in the elderly. Also, there were several contradictions between the reviewed studies, which could be due to several factors, including the novelty of this area of research in environmental gerontology, limited survey tools, or the fact that there is no diversity of theories on the potential combination of effects influencing well-being and activity in aging years. Several personal factors that had an effect on the health and activity of the elderly were identified and led to the realization of a mixed model of effects, which could be an interesting topic for future research.

In this study, a large number of studies written in English language contributed to a broader understanding of the dimensions of active aging. Nevertheless, conducting an integrative review is not a guarantee for finding all relevant articles on the subject of the investigation, as there might have been papers published in other languages than English. A further limitation which restricted the generalizability of the findings was the dearth of any conceptual base in the majority of the studies. Only the study of WHO (2002) sought to conceptualize models for different aspects of active aging.

## Conclusion

This narrative review described the aspects of active aging on a voluminous body of research conducted on the active aging concept using the ecological approach. The results of this study showed that personal characteristics, sociocultural and economic environments, place, and policymaking lead to more health and active life in the elderly (active aging). Also, most reviewed articles proposed that environmental conditions (built, natural, social, cultural, and economic statuses) are among the major factors affecting the elderly’s active aging. However, a few studies claimed that there are no links between environment and active aging. Yet, these articles should pay attention to the effects of the environment in micro, meso, and macro levels, as described in the ecological model. This is while strength, direction, and experience of environmental elements may be different among individuals, communities, and health outcomes in aging years. Future research may focus on the broad topic of environmental gerontology to provide a piece of comprehensive knowledge of the links between environment, aging, health, and activity. Future studies should pay attention to the following factors: conducting qualitative or mixed methods to allow a more detailed exploration; higher levels of collaboration with elderly stakeholders through the research stages and policymaking environment; a more focused consideration of activity participation not only for physical aspects; creating new socioecological models and theories to explain the personal and environmental effects on health and activity; and paying more attention to active aging and relationships between the significant areas of activity participation.

We propose the following definition for the active aging process: *“Active Aging is a process through which an individual tries to maintain the components of health by participating in activities consistent with their objectives, abilities, and opportunities in the community, which can be described as what they want to do and can do, and opportunities to do the activities they enjoy.”*

Also, this study proposes a 5P model, which provides a comprehensive knowledge of diverse aspects of active aging that could be used to benchmark successful active aging and also offers a framework for future research on this topic.

## Data Availability

Not applicable.

## Appendix 1

**Table 3:** Quantitative and mixed studies of active Aging

NO	NAME	TYPE OF STUDY	POPULATION	COUNTRY	DIMENTION OF ACTIVE AGING
1.	[1]	Integrative review (This method includes both qualitative and quantitative studies)	First, 2543 articles Then 76 articles were eligible	Iran	Social well-being, psychological wellbeing, physical health, spirituality and transcendence, and environment and economic security.
2.	[9]	Survey study	Shapefile sources include the United States Census Bureau	USA	Walkability, built environment, physical activity, older adults, objective measures, subjective measures, active aging, GIS, neighborhood, urban health
3.	[33]	multi-method approach (a systematic review and meta-analysis)	100 articles from peer-reviewed and grey literature older adults (≥65 years old)	Australia	Walkability, residential density/urbanization, street connectivity, access to/availability of destinations and services, infrastructure and streetscape, and safety
4.	[23]	Cohort and Followed up study	883 participants aged 55 years and older	USA	Traffic, noise, crime, trash and litter, lighting, and public transportation
5.	[36]	prevalence-based method	17 years of data at age 65 with and without disabilities	Mexico	Disabilities
6.	[37]	Longitudinal study	400 elderly At 60 years of age	Mexico	High blood pressure, type2 diabetes mellitus, cancer, arthritis, osteoporosis, depression, and dementia
7.	[38]	Quantitative approach	235 elderly ranged between 60 and 94 years old	Brazil	Life satisfaction, leisure activities, cigarette smoking, alcohol consumption, practicing of exercises, frequency, activity length, about diet
8.	[39]	Quantitative approach	totalsampleof48adults aged 55 years and over, comprising 4 subsamples of equal numbers (*n* = 12)	Australia	Self-ratings of being active
9.	[40]	Pre- and post-series survey	older adults older than 65 years (*n* = 23)	USA	Longevity, independence, fitness, and engagement
10.	[41]	Scoping study and macro-level analyses	age 60 or 65	Canada	Participation, shopping and obtaining services, active sports, socializing and social participation, car users and non-car-users
11.	[42]	Comparative study	799 community-dwelling older adults between 65 and 74 years old	Canada	Health, participation, and security
12.	[43]	Systematic review and meta-analysis	aged≥65 years	–	Older adults, Active travel, Cycling, Walking, Neighbourhood, Built environment
13.	[45]	Longitudinal Study	aged 50 years and above 307 communities	China	Economic, institutional, and sociodemographic environments paid work, domestic care, participation in community and leisure activities
14.	[46]	Structural Equation Models (SEM)	402 older persons (≥55 years of age)	Singapore	high-density urban neighborhood, well connected street, diversity of land use mix, close proximity to amenities and facilities, and aesthetic environment
15.	[47]	Multiwave study	Over 10 years. In-person interviews were conducted with a stratified random sample of 4162 community dwelling adults aged 65 years and older residing in 5 contiguous counties	USA	Intra-individual (e.g., psychosocial attributes, coping styles, activity accommodations) and extra-individual (e.g., rehabilitation, external supports, and the built, physical, and social environment)
16.	[48]	Cross-sectional studies	Older women (mean age = 69.6; *n* = 136) and women diagnosed with MS (mean age = 46.1; *n* = 173)	USA	Self-efficacy, functional limitations and street connectivity
17.	[49]	Followed up study	age 65–79 years 5218 older	Western Australia	Depression, depressive disorder, mood disorder, mental health, risk factors social context
18.	[52]	Cohort study	1000 participants aged 75, 80 or 85 years	Finland	Wellbeing, disability, environmental and social support, mobility, health behavior and health literacy
19.	[61]	Survey	ages 65–95 (45 female and 55 male)	Turkey	Liveable urban environments, Accessible urban environments, social benefits and opportunities, such as health, social life, environment, well-designed, easy recognizable
20.	[62]	Quantitative approach questionnaire survey	385 older adults aged 60 to 75	Malaysia	Permeability, accessibility, and facilitators to walking
21.	[63]	Cross-sectional interview survey data	4183 older adults (≥60 years)	Thailand	Walkable neighborhood, neighborhood aesthetics, neighborhood service accessibility, neighborhood criminal safety, neighborhood social trust, neighborhood social support, and neighborhood social cohesion. The present study confirms the important role of age-friendly neighborhoods in terms of physical and social environments
22.	[58]	Mixed method approach	117 participants aged 55+ years	China	urban spaces and infrastructure on mobility and well-being
23.	[64]	Mixed-use(research method consists of four phases called as conceptual analysis, data collection about the research area and topic, evaluation of results and discussion)	68 users over 65 years	Turkey	Roads, pollution, safety,insufficiency of maintenance and management, traffic and sociocultural problems
24.	[71]	Cohort study	435 participants aged 65+ years old	USA	High mobility barriers and low transportation facilitators
25.	[74]	Cross-sectional	356 participants 6 to 89 years old	Germany	Intellectual Abilities, Processing Speed, and Processing Robustness
26.	[75]	Population based study	(*N* = 60) aged 55 and over	USA	land use planning and transportation
27.	[76]	Evidence- based	–	Australia	Urban form, parks, walking
28.	[77]	Multilevel regression	546 community-dwelling older adults	USA	Education, Annual household income, Gender, Walking self-efficacy
29.	[80]	Mixed study	97 neighborhood	USA	Quality of life Aging population Spatial demography Heritage city space perception Neighborhood Social networks
30.	[81]	Statistical methodology	1188 older adults	USA	Accessible features (e.g., continuous barrier-free sidewalks and proximity of public transportation)
31.	[78]	multilevel logistic growth curve models	older adults (age 75 +)	USA	Mobility Disability
32.	[82]	Multilevel linear regression analyses	20 selected neighborhoods age (65–74 vs. Z75 years) participants (1750 in total)	Belgium	Walkability and health outcomes
33.	[83]	Survey study	65 years and older 27 Swedish informants interviewed	Sweden	Architecture, Place making, Residential homes
34.	[85]	Data analysis	364 Independently (55–80 years)	Netherland	Walking, Physical environment, Pedestrians, Active transport
35.	[88]	Statistical Analysis	4000 people aged 65 years and over	Hong Kong	Physical and mental components of health, frailty, and mortality. Socioeconomic position, lifestyle factors
36.	[89]	Cross sectional, multilevel design	577 residents (mean age = 74 years) 56 city	USA	Density of places of employment, household density, green and open spaces for recreation, number of street intersections
37.	[90]	Choice-based conjoint analysis	Participants (*n* = 1197)	Belgium	Recreation Public open spaces Park design naturalness, upkeep, walking paths, outdoor fitness equipment/playground, sport field, benches, drinking fountain, peers, mother with children and homeless person
38.	[92]	Survey (questionnaire)	103 participants, ranging in age from 72 to 86 years old	France	Neighborhood satisfaction Well-being
39.	[94]	Cohort study	3144 people born in 1903, 1908, 1913, or 1918	Japan	Age, sex, marital status, baseline functional status, and socioeconomic status, Greenery filled public areas
40.	[95]	A Population-Based Survey	2619 interviews 65 years and over	South Australia	Falls (including slips, trips and falls to the ground)
41.	[96]	Survey (multivariable logistic regression)	4494 elderly Singaporeans (X60 years)	Singapore	Age, gender, ethnicity, education, housing type, living arrangement and social participation) and health (body mass, diabetes and cognitive status)
42.	[97]	Quantitative approach	38,595 elderly persons (≥ 60 years old)	India	Age, tobacco smoking, education, living standard, and other such factors
43.	[99]	Quantitative approach	400 participants Years old+ 60	Iran	Happiness, age, sex, satisfaction, peace, level of activity, self-respect
44.	[98]	quasi-experimental study	Seventy-six older adults aged 60 years and over part	Mexico	Vital Aging, active aging, intervention program, successful aging
45.	[100]	Quantitative approach	–	European countries	labor-market, suicide mortality
46.	[110]	Multivariate logistic regression	1485 participants + 90 years	USA	Dementia, chronic diseases or hospitalizations
47.	[112]	Descriptive-analytic study	379 older adults and 57 managers minimum 60 and maximum 89 years	Iran	Participation and Collaboration of organizations
48.	[113]	A mixed-method sequential explanatory design	all Canadian communities, defined by the municipalities (*N* = 3555)	Canada	1) Describe and compare age-friendly key components of communities across Canada 2) Identify key components best associated with positive health, social participation and health equity of aging adults 3) Explore how these key components foster positive health, social participation and health equity

## Appendix 2

**Table 4:** Qualitative and review studies of active Aging

NO	NAME	TYPE OF STUDY	POPULATION	COUNTRY	DIMENTION OF ACTIVE AGING
1.	[7]	Delphi study	over age 65	UK	Accessible and affordable transportation,housing, healthcare, safety, and community involvement opportunities
2.	[14]	Realist synthesis (is a method of summarizing evidence for public policy)	–	USA	healthy Aging; mobility; neighborhood; public policy
3.	[23]	Systematic review	83 quantitative and qualitative studies	UK	Ethnicity and cultural norms, energy and motivation, sex, age, education, genetic heritage, self-efficacy, and personal financial circumstances, climate, level of pollution, street lighting, traffic conditions, accessibility and appropriateness of services and facilities, socio-economic conditions, aesthetics, pedestrian infrastructure, community life, exposure to antisocial behavior, social network participation, environmental degradation, level of urbanism, exposure to natural settings, familiarity with local environment and others. Recommendations for future research include the need for innovative research methods; involvement of older adults as research collaborators; investigation of wider aspects of the active Aging concept; in-depth assessment of the environmental characteristics of areas; investigation of the pathways leading from environment to health and activity participation.
4.	[31]	A Systematized Review of Qualitative Evidence	36peer-reviewed qualitative studies	Canada	Functional, aesthetic, destination, and safety built characteristics influence physical activity decision-making. Sociodemographic characteristics (age, sex, ethnicity, and socioeconomic status) also impacted the BE’s influence on physical activity
5.	[32]	Synthesizing literature	–	USA	Bonding, bridging and linking capital (Social inclusion)
6.	[34]	Systematic literature review	aged 80 and over	–	Quality of life, subjective well-being, aged, exercise, physical activity
7.	[35]	Grounded theory	–	USA	Body, person and societal level, the person-environment contextual factors
8.	[44]	Content-analyzed	In 33 cities, partners conducted 158 focus groups with persons aged 60 years and older	Global Age-Friendly Cities	Outdoor spaces and buildings; transportation; housing; social participation; respect and social inclusion; civic participation and employment; communication and information; and community support and health services
9.	[50]	Qualitative approach	65 years of age or older	Netherland	Sensory, physical, neural and cognitive functions, housing, safe environment
10.	[51]	Systematic review using a meta-ethnographic approach	-	–	Social, behavioral, biological and psychological factors
11.	[53]	Design	60 years old or above	Hong Kong	Physical, mental and social wellbeing, health, mobility/ability, material circumstances, activities, happiness, youthfulness and living environment
12.	[54]	Literature review	–	Hong Kong	Open spaces, social needs
13.	[55]	Content-analyzed	57 countries		Public health security
14.	[56]	Experience design approach	65 years and over	Australia	Architectural design thinking; user-centric building design; environmental experience design; residential aged care facilities
15.	[57]	Critical review	–	USA	Health, functioning, and social participation, wellbeing
16.	[59]	Literature review	75 article	USA	Safety, microscale urban design elements, aesthetics, and convenience of facilities
17.	[60]	Literature review	–	Czech Republic	Satisfaction, landscape, function
18.	[65]	Qualitative approach	–	USA	Social, economic, demographic, and physical characteristics
19.	[66]	Systematic review	2039 article	USA	Disability Built environment Physical activity
20.	[67]	Concept study	–	Australia	Biological, psychological, behavioral, and social factors include development intensity, land use mix, fine grain economy, adaptability, permeability, streets, contact, visibility and horizontal grain, public realm, movement, green space and water space, landmarks, legibility, comfort, diversity, richness, continuity, contrast, intelligibility, interest, intimacy, openness, rhythm, texture, and human scale.
21.	[68]	Qualitative approach	–	USA	Neighborhood design and safety, housing, transportation, and mobility. Strategies to build capacity for policy change
22.	[69]	Systematic review	aged 50 years and over	Bremen, Germany	Physical activity, Social inequalities
23.	[70]	Review Article	–	–	Active and healthy living; features medical research
24.	[72]	Summative Review	172 review articles aged 65 or older	Australia	Physical activity
25.	[73]	Qualitative approach	over 65 years old	Portugal	Irradiation, Connectivity, Conspicuous, Suitability/Convenience, Readability, Comfort
26.	[79]	Concept study	over 65 years	Poland	Pensions and income. Economy and employment. Health care and other services. Rights of individuals. Housing and communities.
27.	[84]	Literature review	–	USA	Built environment, walking, and health
28.	[86]	Qualitative approach	–	China	Civic participation
29.	[87]	Concept study	–	Herston, Australia	gerontology, public health, environmental psychology, landscape architecture, and urban design personally meaningful outdoor activities, environmental attributes
30.	[91]	Structured review	1464 articles	UK	Health and social services, behavioral determinants, personal determinants, physical environment, social determinants and economic determinants income, health, housing, transport, living in the community, MAori cultural identity, access to facilities and services, attitudes, employment, and opportunities
31.	[93]	Literature Review	48 articles		Poor street condition, Heavy traffic, Public transit line nearby, Housing variable, Environmental barriers, Magnitude of accessibility problems, Housing satisfaction, Usability (Physical environmental aspects), Housing amenities,Satisfaction with home environment, Satisfaction with outdoor environment,Place attachment, Housing accessibility,Housing comfort, Neighborhood quality, Outdoor place,Life Satisfaction, Interior environment, Exterior environment, Residential satisfaction,Psychological wellbeing,Street noise, Safety from traffic, Park density, Train stations, …
32.	[101]	Concept study	–		Social, demographic, financial and political
33.	[102]	Concept study	–	-	Economic justice, satisfying, publicity,
34.	[106]	Concept study	–		Health, participation, Aging, and independence
35.	[103]	Technology-based information, generic ACTION participatory design model	–	West Sweden	Dementia; information and communication technology; participatory design; partnership working; user involvement
36.	[104]	Qualitative approach	–	UK	Inequalities; urban health; older people’s quality of life
37.	[105]	Qualitative research design (Data derived from GPS tracking, travel diaries, brief questionnaires, and semi structured interviews were gathered)	13 people aged from 56 to 87 years	Australia	Choice of transportation and its relation to participation
38.	[107]	Concept study	–		Population health
39.	[108]	Qualitative analysis Focus groups	questionnaire had 57 questions Participants included 18 elderly (aged over 60), five family careers and five professionals	Netherland	Participatory design, patient empowerment and cognitive usability
40.	[109]	Qualitative approach	–	USA	Social, physical, and political residential and business zoning, parks and recreation, transportation, public health, public safety, health services facilities, private sector investment, employment, and taxation
41.	[111]	Qualitative approach	–	Hong Kong	Perspectives of stakeholders—including policy makers, service providers, and elderly learner, quality of life and well-being,
42.	[114]	Literature review	32articles	Australia	‘Age-friendly’, ‘elderly friendly’, ‘livable community’, ‘lifetime neighborhood’ and ‘community for all ages’.
43.	[116]	literature review	–		Public health, human well-being, green infrastructure, urban ecosystem, ecosystem health
44.	[117]	literature review	19 elderly residents (aged 65 years and over)	Australia	Social health; social life
45.	[133]	Qualitative approach	Adults (66–97 years)	Washington	Policy, exercise, obesity, built environment, finite mixture modeling

## Abbreviations

Al: The Author
HB: The Author
PR: The Author
UN: United Nation
WHO: World Health Organization
