# Correction to: Comprehensive 5P framework for active aging using the ecological approach: an iterative systematic review

**DOI:** 10.1186/s12889-020-8227-6

**Published:** 2020-01-23

**Authors:** Azadeh Lak, Parichehr Rashidghalam, Phyo K. Myint, Hamid R. Baradaran

**Affiliations:** 1 Faculty of Architecture and Urban Planning, Shahid Beheshti University, Tehran, 1983963113 Iran; 2 Ageing Clinical & Experimental Research Team, Institute of Applied Health Sciences, University of Aberdeen, Aberdeen, UK; 3 Ageing Clinical & Experimental Research Team, Institute of Applied Health Sciences, University of Aberdeen, Aberdeen, UK; 4 Department of Epidemiology, School of Public Health, Iran University of Medical Sciences, Tehran, Iran

**Correction to: BMC Public Health (2020) 20:33**

**https://doi.org/10.1186/s12889-019-8136-8**

It was highlighted that the original article [1] contained a spelling mistake in the name of Hamid R. Baradaran. This was incorrectly captured as Bradaran. The original article has been updated.
